# Recurrent fetal truncus arteriosus associated with PMM2‐CDG

**DOI:** 10.1002/jmd2.12193

**Published:** 2021-03-30

**Authors:** Filipa Malheiro, Manuela Silva, Carlos Macedo, Carla Ramalho

**Affiliations:** ^1^ Department of Obstetrics Centro Hospitalar e Universitário de São João Porto Portugal; ^2^ Department of Obstetrics‐Gynecology and Pediatrics, Faculty of Medicine University of Porto Porto Portugal; ^3^ Department of Obstetrics‐Gynecology Hospital Dr. Nélio Mendonça Madeira Portugal; ^4^ i3S Instituto de Inovação e Investigação em Saúde Porto Portugal

To the Editor:

The recent article “Vascular ring anomaly in a patient with phosphomannomutase 2 deficiency: a case report and review of the literature” published in *JIMD Reports* by Quian et al suggests that congenital defects of glycosylation (CDG) should be part of the differential diagnosis in patients with cardiac malformations.^1^ We believe our case is relevant in this context.

A nonconsanguineous couple, with a history of two medical terminations of pregnancy following a truncus arteriosus diagnosis, was counseled at our Prenatal Diagnosis Center. Both were spontaneous pregnancies with no complications until the ultrasound diagnosis of a truncus arteriosus malformation.

In the first pregnancy, the pathology exam confirmed the ultrasound diagnosis of type 2 truncus arteriosus and also identified a probable cerebellum hypoplasia.

In the second pregnancy, the pathologic findings were also compatible with a type 2 truncus arteriosus and the microscopy exam suggested an irregularity of the vermis of the cerebellum.

The recurrent diagnosis motivated the whole exome gene sequencing in the second fetus (the genetic study of the first fetus is ongoing) that identified two heterozygous variants in the phosphomannomutase 2 (PMM2) gene: c.415G>Ap.(Glu139Lys) and c.422G>A p.(Arg141His). The variant c.415G>Ap.(Glu139Lys) was detected in the mother and the variant c.422G>A p.(Arg141His) in the father. No other variants were found in this gene.

PMM2 pathogenic variants are the most common cause of CDG, which is associated with neurodevelopmental disability and affects multiple organ systems including cerebellar abnormalities. However, the association with congenital heart defects (CHDs) is unclear. Quian et al has undertaken a literature review and found a prevalence of 1.5% (21.4% truncus arteriosus) of CHDs in 960 patients with PMM2‐CDG vs a prevalence of 0.9% (1% of truncus arteriosus) reported in the general population.

Accordingly, screening for other anomalies is of the utmost importance. The identification of an isolated defect or the presence of other concomitant anomalies cannot make or exclude the diagnosis.

The identification of the PMM2 gene variants in this couple allowed us to inform them of a risk of 25% of CDG in every pregnancy and gives them a possibility of an earlier diagnosis in case of a spontaneous pregnancy or access to preimplantation genetic testing for monogenic disorders (PGT‐M).

Here we present two more PMM2‐CDG patients with cardiac malformations adding to the 14 described in the literature. Five of these had the same gene variants as the subjects. Our findings reinforce the importance of including CDG in the differential diagnosis of patients presenting with syndromal CHDs.

## CONFLICT OF INTEREST

The authors declare no potential conflict of interest.

## AUTHOR CONTRIBUTIONS

Filipa Malheiro: data collection, drafting the article; Manuela Silva: data collection, revising the article; Carlos Macedo: data collection, revising the article; C. Ramalho: conception, revising the article.

## PATIENTS CONSENT STATEMENT

This article does not contain any studies with human or animal subjects performed by the any of the authors.

## References

[1] Quian Z , Van den Eynde J , Heymans S , Mertens L , Morava E . Vascular ring anomaly in a patient with phosphomannomutase 2 deficiency: a case report and review of literature. JIMD Rep. 2020;56:27‐33. 33204593 10.1002/jmd2.12160 PMC7653259

